# Homocysteine Level and Risk of Abdominal Aortic Aneurysm: A Meta-Analysis

**DOI:** 10.1371/journal.pone.0085831

**Published:** 2014-01-21

**Authors:** Hui Cao, Xinhua Hu, Qiang Zhang, Jun Li, Junpeng Wang, Yang Shao, Bing Liu, Shijie Xin

**Affiliations:** Department of Vascular and Thyriod Surgery, the First Affiliated Hospital of China Medical University, Shenyang, China; University of Louisville, United States of America

## Abstract

**Objectives:**

Previous studies have reported inconsistent findings regarding the association between elevated plasma homocysteine (Hcy) levels and abdominal aortic aneurysm (AAA). We investigated this association between Hcy levels in patients with AAA and unaffected controls by conducting a meta-analysis and systematic review.

**Methods:**

We conducted a systematic literature search (up to August 2013) of the PubMed database and Embase. We selected observational studies that evaluated Hcy levels in subjects with AAA compared to unaffected controls. Criteria for inclusion were the assessment of baseline Hcy and risk of AAA as an outcome. The results were presented as odd ratio (OR) and corresponding 95% confidence intervals (CI) comparing AAA patients to the control subjects.

**Results:**

7 studies with 6,445 participants were identified and analyzed. Overall, elevated plasma Hcy was associated with an increased risk of AAA (3.29; 95% CI 1.66–6.51). The pooled adjusted OR from a random effect model of only men participants in the AAA compared with the control group was 2.36 (95% CI 0.63–8.82).

**Conclusion:**

This meta-analysis and systematic review suggested that Hcy significantly increased the risk of AAA.

## Introduction

Abdominal aortic aneurysm (AAA) is an abnormal dilatation of the abdominal aorta, exceeding the normal aortic diameter by at least 50%. Aortic aneurysms do not tend to cause any symptoms and are often diagnosed incidentally on physical examination or imaging investigations for other unrelated conditions [1], [2]. Screening demonstrates that disease prevalence ranges from 1.3% (45–54 years of age) to 12.5% in men (75–84 years of age), and in women from 0% in the youngest to 5.2% in the oldest age groups [3]. Currently there are no recognized treatments to diminish aneurysm progression at an early stage and a pharmacological treatment that targeted the processes underlying aortic dilatation [4].

Homocysteine (Hcy) is a branch-point intermediate of methionine metabolism, which can be further metabolised via two alternative pathways: degraded irreversibly through the transsulfuration pathway or remethylated to methionine by the remethylation pathway [5], [6]. Vitamin B and folate are major determinants of Hcy metabolism and supplementation with vitamin B and folic acid has been shown to be effective in normalizing Hcy levels [7]–[10]. Elevated total plasma level of Hcy has been shown to correlate strongly with coronary artery disease [11], [12], stroke [13], [14], fracture [9], [15], venous thrombosis [13], and may increase the risk of retinal artery [16] and vein occlusion [17] and nonarteritic anterior ischemic optic neuropathy [18]. Several studies reported that the elevated Hcy concentrations were identified as a potentially modifiable risk factor for abdominal aortic aneurysms [5], [6], [19]-[23]. Based on the previously published clinical evidence, a clinical review demonstrated there is evidence for an association between Hcy and AAA [24]. However, it is not strong enough to conclude that it plays a causal role in the pathogenesis of AAA. In the current study, we performed a meta-analysis to assess the relationship between Hcy and AAA in the literature. The aim of the current meta-analysis and systematic review was to quantitatively evaluate findings from observational studies on the plasma level of Hcy and the incidence of AAA.

## Methods

This study was conducted in accordance with the ‘preferred reporting items for systematic reviews and meta-analyses'(PRISMA) guidelines. No protocol exists for this meta-analysis.

### Search strategy

We conducted a PubMed database and Embase search (up to August 2013) for studies assessing the association between AAA and Hcy. Papers could be published in English and Chinese. Potentially relevant studies were identified by various combinations of the following search terms: abdominal aortic aneurysm, hyperhomocysteinemia, homocysteine, folate, folic acid, vitamin B_6_, and pyridoxine. In addition, we also manually searched the reference lists to detect additional eligible studies.

### Study selection

Studies satisfying the following criteria were included in the observational meta-analysis: 1) providing raw data dealing with AAA and Hcy level and the risk of AAA; 2) AAA was defined as computed tomography (CT) and ultrasonographically proven infrarenal aortic diameter ≥30 mm); 3) Hcy level was measured at baseline using a technique standardized in each study; 4) providing adjusted odd ratio (OR) and the 95% confidence interval (CI) comparing the Hcy level of AAA to the control group. Exclusion criteria were: reviews, letters, case-reports, animal studies or the participants in a highly selected disease.

### Data extraction and quality assessment

Two reviewers (Xinhua Hu and Qiang Zhang) independently extracted the data according to predefined criteria using a “AAA associated with Hcy extraction form” developed specifically for the review. The OR and 95% CI were extracted. When both crude and adjusted OR were provided, we used the most fully adjusted OR for all the included studies. We also extracted the following items from each individual study: author; year of publication; the country of study; the sample size; gender; and the mean age or age range of participants; Hcy level; case and control number. Where discrepancies were identified, reviewers resolved these by discussion.

Quality assessment that was performed with consideration of the following aspects followed the Newcastle-Ottawa Scale [25]: 1) Selection (4 scores): is the case definition, representativeness of the cases, selection of controls, and definition of controls. 2) Comparability (2 scores): select the most important factor, the criteria could be modified to indicate specific control for as second important factor. 3) Exposure (3 scores): ascertainment of exposure, same method of ascertainment for cases and controls, and non-response rate.

### Statistical analysis

The OR was used as the common measure across studies. Data analysis used multivariate-adjusted OR and 95% CI. If the publications reported separately OR for smoker, we chose the separate OR estimates for the different items comparing AAA to the control from individual study. Before pooling the data, adjusted OR from each study was converted to their logOR to stabilize the variances and to normalize the distributions. The standard errors (SEs) for logOR were calculated from reported 95% CI. Homogeneity of OR across studies was assessed by using the Cochrane Q statistic (p<0.10 was considered indicative of statistically significant heterogeneity) and I^2^ statistic (values of less than 40% as “heterogeneity might not be important ” and of more than 75% as “considerable heterogeneity”, based on the suggestion of the Cochrane Handbook for Systematic Review of Interventions) [26]. The pooled OR was computed using either fixed-effects models or, in the presence of heterogeneity, random-effects models [27].

We constructed a funnel plot with logOR and their SEs of logOR using AAA by visual inspection to assess the potential publication bias. Potential publication bias was also assessed by both Begg' rank correlation test [28] and Egger linear regression test at the p<0.10 level of significance [29]. Finally, sensitivity analysis was used to investigate the influence of a single study on the overall risk estimate, and was carried out by sequentially omitting one study at each turn with the metaninf algorithm in STATA. All analysis was performed using STATA version 12.0 statistical software (Stata Corp LP, College Station). A p value <0.05 was considered as statistically significant.

## Results

### Literature search

**Figure 1:**
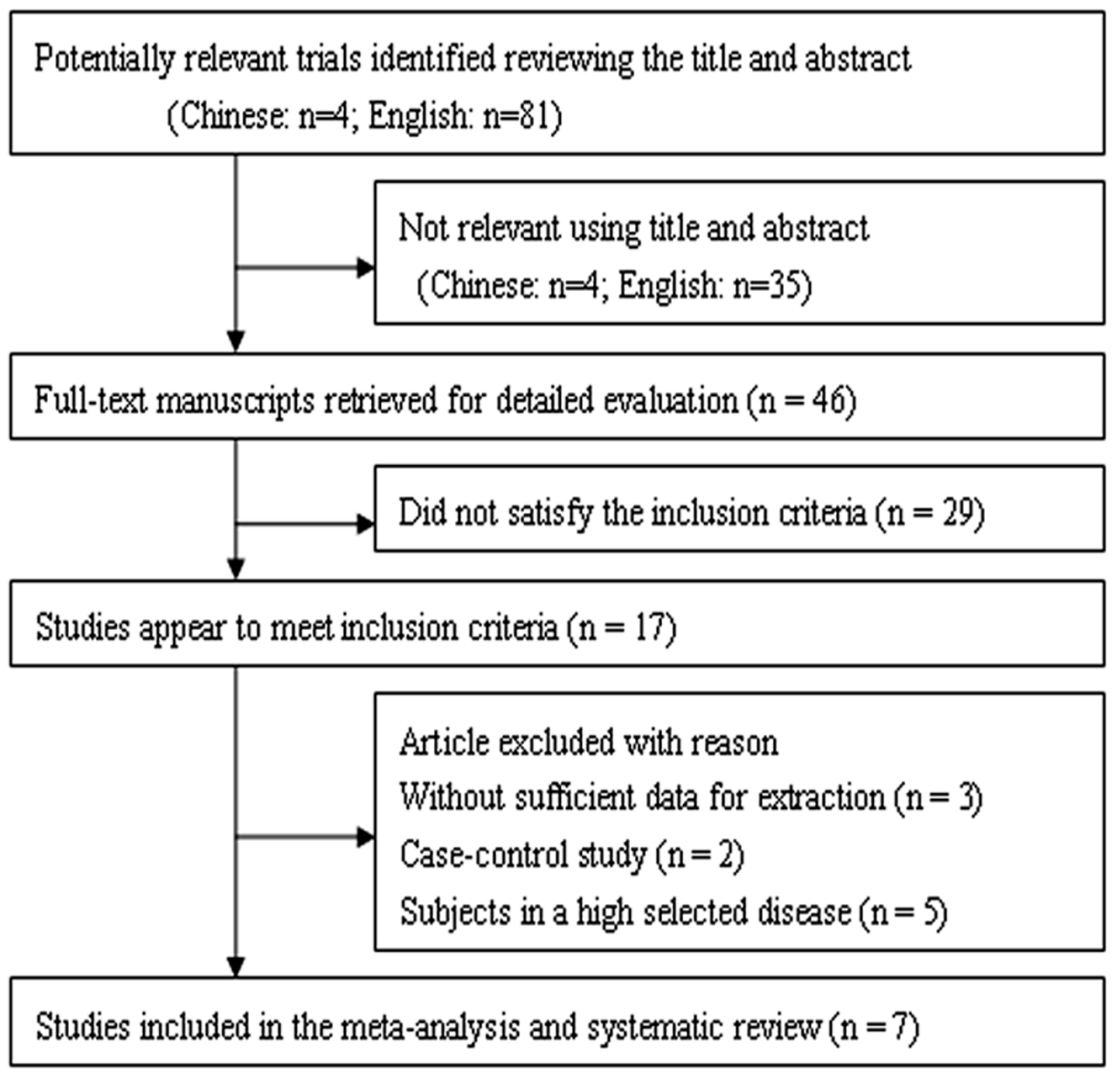
Flow chart of study selection process for meta-analysis.

After the application of search strategy, a total of 85 potentially relevant citations were identified in our initial literature search. After reviewing the full-texts, only 7 studies met the inclusion criteria [5], [6], [19]–[23]. Analysis was carried out on 1410 patients with an AAA (infrarenal aortic diameter ≥30 mm) and 5035 age and sex matched controls. A flow chart showing the study selection is presented in Fig 1. No additional studies were identified through our hand search of references from published studies.

### Baseline characteristics and quality assessment

Characteristics of the included studies were listed in Table 1. The qualities of the included studies were listed in Table 2.

**Table 1:** Summary of clinical studies included in meta-analysis.

Study/year	Country	Design	Subjects	Age/range Mean(SD)	Male	Hcy levels (Mean±SD), µmol/L		OR (95% CI)	Adjustment for covariates
			cases/controls	cases/controls	cases/controls	AAA patients	controls		
Wong YYE (2013)	Australia	case-control study	318/3930	77.7±4.1/76.5±3.6	318/3930	15.1±6.1	13.2±5.1	1.45(1.10–1.91)	Age, sex,smoking, hypertension, BMI, aortic diameter, dyslipidemia, diabetes, Cardiovascular disease, hsCRP
Giusti B (2008)	Italy	case-control study	423/423	73.5(40–94)/72(41–94)	376/366	15.3(6.7–144.3)	14.1(6.1–94.9)	1.1(1.01–1.20)	Age, sex,smoking, hypertension, dyslipidemia, diabetes mellitus, and COPD
Peeters AC (2007)	Netherlands	case-control study	88/88	69(45–85)/67(44–83)	81/81	quartil 9.33,11.8,15.1	N	0.78(01.7–3.62)	Age, sex,smoking, hypertension, dyslipidemia, concentration of creatinine, vitamin B_6_, B_12_, and folate
Sofi F(2005)	Italy	case-control study	438/438	74(40–94)/73.4(48–84)	397/391	16.2(7.3–93.6)	11(6–24.6)	7.8(4.60–13.20)	Age, sex,smoking, hypertension, aortic diameter, dyslipidemia
Warsi AA (2004)	UK	case-control study	38/36	70(53–79)/66(48–79)	35/21	19.4	10.9	9.75(2.17–43.77)	Age, sex,smoking, hypertension, aortic diameter, dyslipidemia, diabetes, cardiac disease, stroke
Spark JI (2003)	Australia	case-control study	47/60	N	N	17.9(14.8–20.8)	8.5(7.0–11.8)	15.9(4.96–51.00)	Age, sex,smoking, hypertension, aortic diameter, dyslipidemia,diabetes, cholesterol
Brunelli T (2000)	Italy	case-control study	58/60	69.3±6.7/67.6±7.0	58/60	15.7±6.5	9.6±3.9	6(1.23–29.6)	Age, smoking, hypertension, aortic diameter, diabetes mellitus, Hypercholesterolemia, Hypertriglyceridemia

Abbreviations: Body mass index, BMI; odd ratio, OR; not provide, N; chronic obstructive pulmonary disease, COPD; high-sensitivity C-reactive protein, hsCRP.

**Table 2:** The Newcastle-Ottawa Quality Assessment Scale for case–control studies.

Study	Selection	Comparability	Outcome	Summary
Wong YYE (2013)	4	2	2	8
Giusti B (2008)	4	2	2	8
Peeters AC (2007)	4	2	3	9
Sofi F (2005)	4	2	3	9
Warsi AA (2004)	4	2	3	9
Spark JI (2003)	4	2	2	8
Brunelli T (2000)	4	2	3	8

### Association of plasma Hcy with all AAA

**Figure 2:**
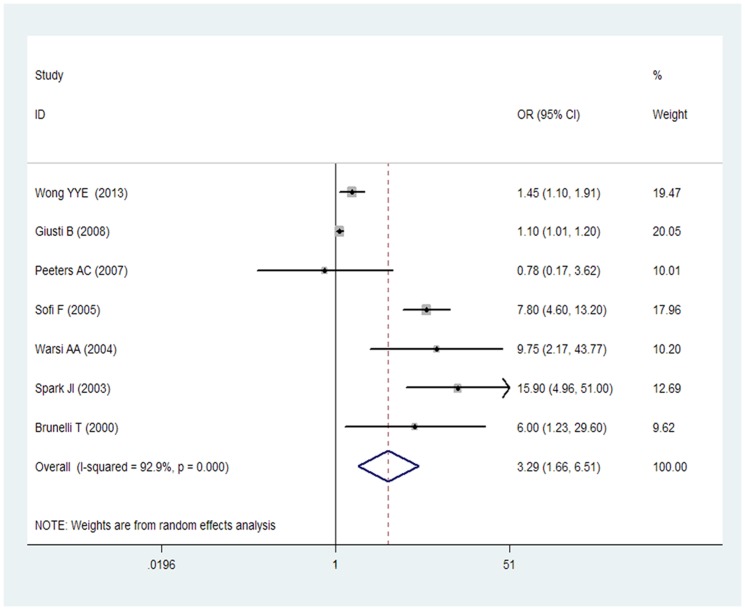
OR and 95% CI from the included studies of plasma Hcy level and AAA.

7 studies [5], [6], [19]–[23] reported AAA and Hcy. The total number of participants included in this meta-analysis was 6445 with 1410 total AAA patients. As shown in Fig 2, the pooled adjusted OR from a random effect model for all studies combined for the Hcy levels that was associated from a random effect model of participants with AAA was 3.29 (95% CI 1.66–6.51), with evidence of significant heterogeneity (I^2^ = 92.9%; p = 0.000). The pooled OR from a fixed-effect model was 1.21 (95% CI, 1.11–1.31).

**Figure 3:**
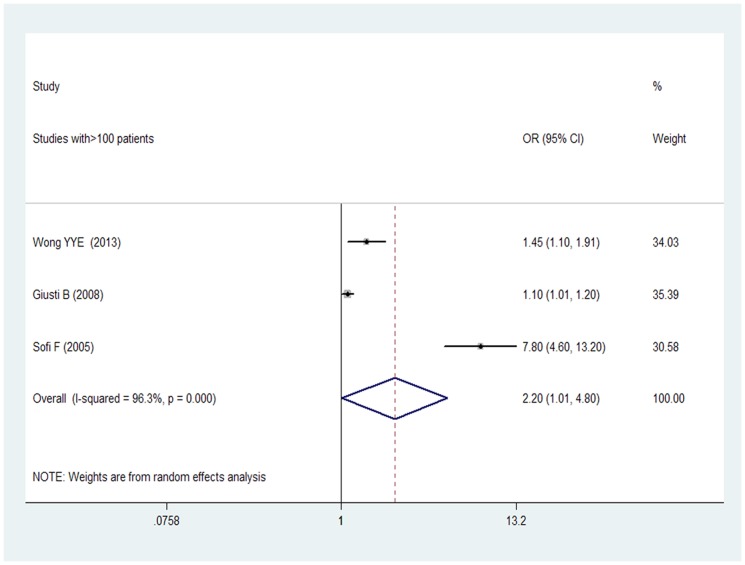
OR and 95% CI from the studies with >100 patients of plasma Hcy level and AAA.

**Figure 4:**
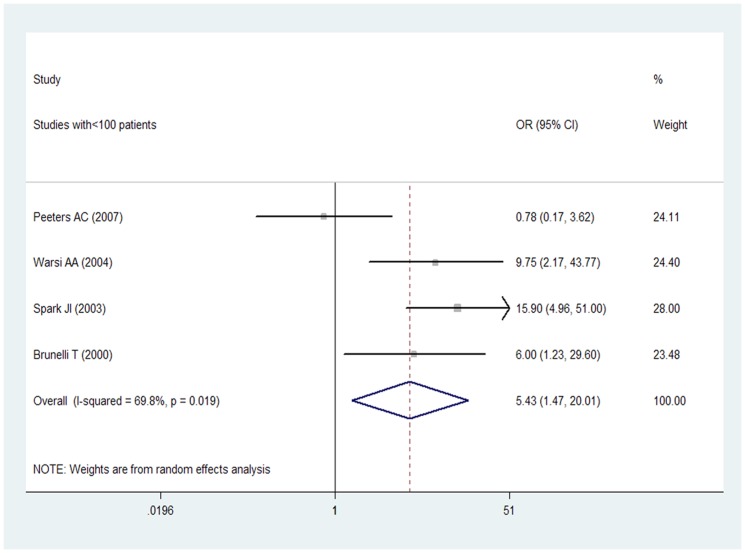
OR and 95% CI from the studies with <100 patients of plasma Hcy level and AAA.

Of the 7 included studies 3 actually had more than 100 subjects within each cohort [5], [6], [20], and the pooled adjusted OR from a random effect model for the 3 studies combined for the Hcy levels that was associated from a random effect model of participants with AAA was 2.20 (95% CI 1.01–4.80), with evidence of significant heterogeneity (I^2^ = 96.3%; p = 0.000) (Fig 3). 4 studies had less than 100 subjects [19], [21]–[23], and the pooled adjusted OR from a random effect model for the 4 studies combined for the Hcy levels that was associated from a random effect model of participants with AAA was 5.43 (95% CI 1.47–20.01), with evidence of significant heterogeneity (I^2^ = 69.8%; p = 0.019) (Fig 4).

**Figure 5:**
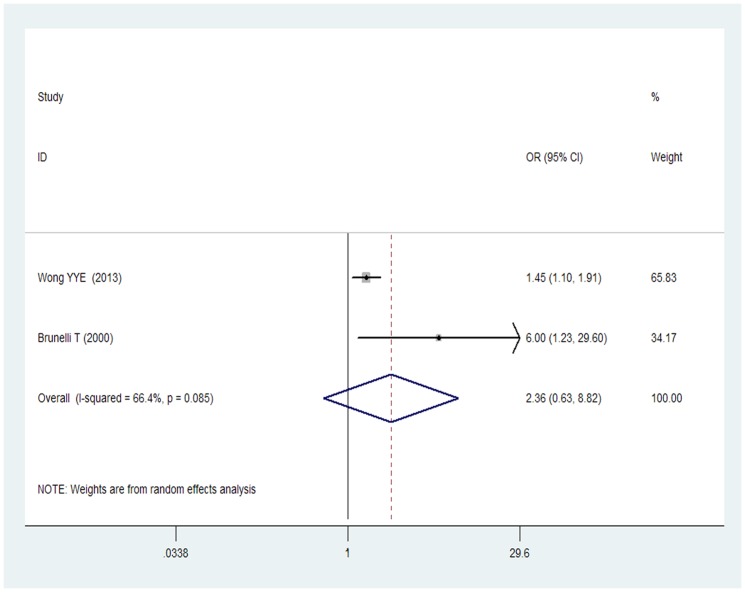
OR and 95% CI from the included studies of plasma Hcy level and AAA by men.

As shown in Fig 5, the pooled adjusted OR in two studies [5], [23] from a random effect model of participants with AAA in the high Hcy group compared with the normal Hcy concentrations group only men was 2.36 (95% CI 0.63–8.82), with evidence of significant heterogeneity (I^2^ = 66.4%; p = 0.085).

### Sensitive analysis on AAA

**Figure 6:**
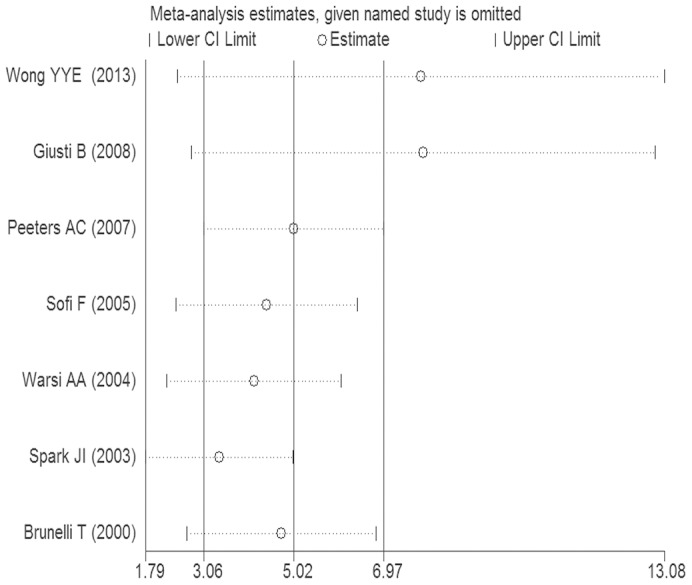
OR and 95% CI by omitting each study from the included studies of plasma Hcy and AAA.

As shown in Fig 6, in the metainf analysis, there was little change in the quantitative summary measure of OR or 95% CI.

### Assessment of publication bias

Evidence of publication bias for studies reporting adjusted OR of AAA was noted in asymmetrical funnel plot on visual inspection, and Egger' s linear regression test (p = 0.049), but not in the Begg's rank correlation test (p = 1.000). There was no evidence of publication bias for studies reporting adjusted OR of abdominal aortic diameter, this was further confirmed by Begg's rank correlation test (p = 0.806) and Egger's linear regression test (p = 0.136).

## Discussion

The findings of the current meta-analysis provided evidence that Hcy was a predictor of increased AAA risk. Subjects with elevated Hcy increased risk of AAA (OR 3.29; 95% CI 1.66–6.51).

The major environmental exposures associated with AAA development include sex, advancing age, cigarette smoking, dyslipidaemia, and hypertension [30]. In the current meta-analysis, male was not associated with a markedly increase AAA risk (OR 2.36; 95% CI 0.63–8.28). However, due to the limited included study number and low proportion (376 of 1410) of AAA, the apparent lack of association with AAA and gender might be not convincing.

Pathological characterization of AAA includes intimal atheroma, infiltration of inflammatory cells, such as macrophages, T lymphocytes, neutrophils and dendritic cells and activated platelets into the aortic wall. These early events are followed by extracellular matrix (ECM) destruction and remodelling, vascular smooth muscle cell (VSMC) depletion and dysfunction [31]. Experiments had demonstrated that Hcy stimulates chemokine and cytokine secretion from cultured human monocytes and has been implicated in suppressing regulatory T-cell function [32]. A recent interesting study by Liu Z et al., within an angiotensin II induced AAA mouse model suggests that hyper-Hcy exaggerates adventitial inflammation, promoting AAA [33]. Hcy induces the synthesis of serine elastase in arterial smooth muscle cells, causing elastolysis by degradation of the extracellular matrix and release of reactive oxygen species, which are implicated in AAA pathogenesis [34], [35]. Whether Hcy plays a role in aneurysm formation and/or in aneurysm expansion or Hcy is simply a marker of the condition need to be investigated.

Plasma Hcy levels are also influenced by several factors such as genetic factors [36], [37], plasma folate and vitamin B_12_ concentrations. Selhub and colleagues have suggested that inadequate plasma concentrations of one or more B vitamins are contributing factors in approximately two thirds of all cases of hyperhomocysteinaemia and that vitamin supplementation can normalise high homocysteine concentrations [7]. As lack of well-designed RCTs, we were unable to perform a meta-analysis of the clinical effects of Hcy-lowering therapy among the limited studies because of the in consistent outcomes. Hcy-lowering therapy by folic acid, vitamins B_6_ and B_12_ supplement will help to reduce rates of AAA or not, the answer maybe urgent in the development of interventions to prevent AAA. More well-design RCTs are needed to test the effects of Hcy-lowering therapy on the AAA incident.

Although we only included the case-control studies in the analysis, there are still several potential limitations. First, because of the cross-sectional design, the studies are unable to determine if the altered parameters are causally related to the presence of AAA. Second, we only included the study in English and Chinese, and some relevant studies might be not included in the review. There was a low risk of publication bias in studies of Hcy concentrations in relation to all AAA, as suggested by asymmetrical funnel plot on visual inspection and Egger's linear regression test (p = 0.049). Third, there was significant heterogeneity (I^2^ = 92.9% in all AAA) among the included studies. Another potential source of heterogeneity was the lack of uniform definition of subjects. Forth, a major limitation was the possibility of uncontrolled confounding, and the individual studies did not adjust for potential risk factors in a consistent way. A wide spectrum of diseases was associated with elevated plasma Hcy concentrations, such as cardiovascular disease, stroke and cognitive impairment. These diseases were also associated with AAA; some residual confounding factors still affected the results. None of the analyzed studies seems to have adjusted Hcy levels for its most important covariate, glomerular filtration rate (GFR), and this is a major problem for the outcome. The lack of adjustment for these confounding factors might have resulted in a slight over estimation of the OR. Finally, the studies of Wong YYE and the Brunelli T contained only men, and that may make the findings have only limited relevance to AAA in women [5], [23].

## Conclusions

In conclusion, this meta-analysis and systematic review provided evidence that Hcy was associated with AAA. Whether this link is causal remained to be clarified further by future studies. More well-design RCTs on the effect of Hcy-lowering therapy on the prevention or treatment of AAA would be helpful to clarify this question.

## Supporting Information

Checklist S1 **PRISMA checklist.**
(DOC)
